# Identifying mortality risks in patients with opioid use disorder using brief screening assessment: Secondary mental health clinical records analysis

**DOI:** 10.1016/j.drugalcdep.2016.04.036

**Published:** 2016-07-01

**Authors:** Karolina Magda Bogdanowicz, Robert Stewart, Chin-Kuo Chang, Johnny Downs, Mizanur Khondoker, Hitesh Shetty, John Strang, Richard Derek Hayes

**Affiliations:** a King’s College London, Institute of Psychiatry Psychology and Neuroscience, London SE5 8AF, UK; b University College London, Institute of Epidemiology and Health, UK; c South London and Maudsley NHS Foundation Trust, London SE5 8AZ, UK

**Keywords:** Opioids, Heroin, Treatment, Mortality, Risk assessment, Suicide, Overdose, Injecting

## Abstract

**Background:**

Risk assessments are widely used, but their ability to predict outcomes in opioid use disorder (OUD) treatment remains unclear. Therefore, the aim was to investigate if addiction-specific brief risk screening is effective in identifying high mortality risk groups and if subsequent clinical actions following risk assessment impacts on mortality levels.

**Methods:**

Opioid use disorder (OUD) patients were identified in the South London and Maudsley Case Register. Deaths were identified through database linkage to the national mortality dataset. Cox and competing-risk regression were used to model associations between brief risk assessment domains and all-cause and overdose mortality in 4488 OUD patients, with up-to 6-year follow-up time where 227 deaths were registered. Data were stratified by admission to general mental health services.

**Results:**

All-cause mortality was significantly associated with unsafe injecting (HR 1.53, 95% CI 1.10–2.11) and clinically appraised likelihood of accidental overdose (HR 1.48, 95% CI 1.00–2.19). Overdose-mortality was significantly associated with unsafe injecting (SHR 2.52, 95% CI 1.11–5.70) and clinically appraised suicidality (SHR 2.89, 95% CI 1.38–6.03). Suicidality was associated with a twofold increase in mortality risk among OUD patients who were not admitted to mental health services within 2 months of their risk assessment (HR 2.03, 95% CI 1.67–3.24).

**Conclusions:**

Diagnosis-specific brief risk screening can identify OUD patient subgroups at increased risk of all-cause and overdose mortality. OUD patients, where suicidality is evident, who are not admitted into services are particularly vulnerable.

## Highlights

• Prompt identification of those at risk is key.
• We examine clinical appraisal of patient risks and mortality in 4488 opioid dependent patients.
• Addiction-specific risk assessment is useful in predicting mortality.
• Non-admission of patients where suicidality is evident increases mortality.

## 1 Introduction

People dependent on heroin or other opioids are up to 14 times more likely to die than their peers (Darke and Ross, 2002). Worldwide, an estimated 69,000 people die from opioid overdose (accidental or deliberate) each year (World Health Organisation (WHO), 2014). In England and Wales, more than 1700 deaths registered in 2014 (53% of all deaths from drug poisoning) involved an opiate drug (Office For National Statistics (ONS), 2015). Assessing and managing risks is a paramount element of care planning and treatment provision to people with drug dependence, particularly in opioid dependence (Department of Health (DOH), 2007). Assessment of risks within the addictions services should be substance misuse specific, prioritizing directly related risks such as overdose, poly-drug use, suicide and/or unsafe injecting practices (National Treatment Agency for Substance Misuse, 2006a, National Treatment Agency for Substance Misuse, 2006b).

The effectiveness of risk assessment tools in predicting mortality in mental healthcare is unclear. Wand, 2012 reported inability to conduct a systematic review due to paucity of studies evaluating the effectiveness of risk assessments, and found little evidence to conclude whether risk assessments are effective in relation to self-harm or suicide reduction. Studies attempting to identify individuals who are likely to die by suicide have been largely unsuccessful primarily due to its low prevalence, even within high-risk groups (Harriss and Hawton, 2005, Kapur, 2005). A recent study of people receiving secondary mental healthcare reported that the level of clinically appraised risk of self-neglect (but not suicide or violence) predicted all-cause mortality, but the study did not stratify results by diagnosis or examined cause-specific mortality (Wu et al., 2012). Given the differences in aetiology, symptoms, care provision and risk factors between mental health diagnostic groups, it is important to investigate these separately as advised by the NTA (2006a). Therefore, the aim of the current study was to determine if addiction-specific brief risk assessment completed for opioid use disorder patients is effective in predicting risks of all-cause and overdose mortality; to investigate mortality levels in patients clinically appraised as displaying suicidality, increased likelihood of accidental overdose and unsafe injecting practices; and to determine if associations between clinically appraised risks and mortality differs depending on subsequent clinical actions such as admission to secondary mental health services and the type of opioid substitution therapy (OST) prescribed.

## 2 Methods

### 2.1 Study setting

South London and Maudsley NHS Foundation Trust (SLaM) is one of the largest secondary mental healthcare services in Europe, currently providing comprehensive mental healthcare and addiction service to a catchment population of approximately 1.2 million residents across seven ethnically and socially diverse, high population density boroughs of south London. SLaM addiction services have used electronic health records (EHRs) since April 2008. In the same year, at the SLaM NIHR Biomedical Research Centre for Mental Health, the Clinical Record Interactive Search (CRIS) was developed. CRIS uses EHRs in a de-identified format, allowing researchers to search and retrieve complete case records for analytical purposes. There are currently more than 260,000 patients represented on the system. CRIS was approved as a dataset for secondary analysis by Oxfordshire Research Ethics Committee C (reference 08/H0606/71+5), and its protocol is described in detail elsewhere (Perera et al., 2016, Stewart et al., 2009).

### 2.2 Inclusion criteria

Diagnoses in SLaM are coded in accordance with the 10th edition of the World Health Organization International Classification of Diseases (ICD-10; WHO, 1993). This study cohort comprised SLaM patients who were diagnosed with an ICD-10 F11 primary or secondary opioid use disorder (OUD) between 1st April, 2008 to 31st March, 2014 (inclusive), and who had at least one item completed on the Brief Risk Scale Assessment—Addiction (BRSA-A) during the observation period. Diagnoses were derived from their designated SLAM EHR structured fields and from free-text fields using Natural Language Processing (NLP). The NLP application for ‘diagnosis’ sought to extract any text strings associated with a diagnosis statement in order to supplement the existing structured fields. The performance of the ‘diagnosis’ NLP application was evaluated formally elsewhere (Sultana et al., 2014). In the SLaM case register, OUD is the second most frequently diagnosed substance use disorder after alcohol use dependence (Hayes et al., 2011).

### 2.3 Main outcome measures

#### 2.3.1 All-cause mortality

The main outcome in this study was all-cause mortality in individuals with primary or secondary diagnosis of OUD, within the period 1st April, 2008 to 31st March, 2014. Every death in the UK is reported to the Office for National Statistics General Records Office, which is then conveyed to the NHS Care Records Service and available to all NHS organisations. Majority of deaths are registered with ONS within five days and SLaM mortality updates are performed on a monthly basis. This allowed us to establish deaths within the observation period, for both active and inactive SLaM patients. The full procedure for identifying and confirming SLaM patient deaths has been described elsewhere (Chang et al., 2010).

#### 2.3.2 Cause-specific mortality

Additionally, 68.7% of all those who died had death certificate information. This information allowed us to establish cause-specific mortality, and more specifically coding for overdose mortality. Fatal overdoses included a combination of both intentional (i.e., suicide) and unintentional (i.e., drug poisoning) overdose deaths, with ICD-10 codes X409-X450, Y120, Y125 and F119 sub-classified as such. The relationship between heroin overdose and suicide is problematic due to ambiguous circumstantial information and unclear intent (Cantor et al., 2001), therefore for these analyses, we grouped suicide by overdose and fatal drug poisonings into one group. The cause of death information is based on a static ONS-CRIS data linkage and is more likely to reflect a proportion of delayed as well as recent occurrences of deaths within the ONS (ONS, 2011), resulting in the 31% missing causes of death in our cohort.

### 2.4 Exposures

The main exposures of interest in this study were patients’ risks of suicidality, likelihood of overdose and injecting practices. These three risk domains were recorded using the Addiction Brief Risk Scale Assessment (BRSA-A) (described below) in patients with OUD

In addition to the main exposures of interest, a number of other covariates were considered as potential confounders. Patients’ risks associated with violence, health, social variables, and service use were also recorded on the BRSA-A. Ethnicity and gender are routinely recorded on SLaM electronic patient records in their designated fields. Age was calculated on the date on which individuals received their first BRSA-A assessment within the observation period. Ethnic group classifications were condensed to “White British”, “Other White background”, “African, Caribbean and other black background”, and “Mixed, unknown and other”. Area-level deprivation was established by linking the patient’s residential postcode to the UK Census data projected for 2007 in lower super output area units. The full procedure for measuring level of deprivation is described elsewhere (Hayes et al., 2012). Homelessness variable was established by merging information from area-level deprivation and homelessness/unstable housing item on the BRSA-A scale. Information on patient admissions to a SLaM secondary mental health service in the two-month period after BRSA-A assessment was also extracted. This information included general admissions to SLaM, and information on prescription of opioid substitute treatment (OST) medication (i.e., buprenorphine, methadone, Suboxone [buprenorphine/naloxone]) in the 2-month period after BRSA-A completion. Information extracted included both inpatient and outpatient community service admissions/prescriptions in a 60-day (two months) observation period after the BRSA-A completion.

### 2.5 Risk assessment instrument

The BRSA-A is a compulsory target for the addictions clinical team on all active cases. This risk measure was developed by SLaM clinicians to encourage identification and formal recording of risk areas specific to substance misuse patients; these are then used in their care planning. BRSA-A should be completed for each service user at the point of referral, as part of the service user’s initial assessment when he/she first comes into contact with SLaM services. The completion of the BRSA-A assists in informing clinical staff whether a full risk screen is then required (SLaM, 2011).

The BRSA-A includes twenty-seven binary items (0 = no risk; 1 = risk detected). These individual items have been sub-classified into seven risk domains: suicidality, accidental overdose, injecting practices, violence, health, social, and service use. The full list of individual BRSA-A items and their classified risk domains are presented in Table 1. For analytical purposes we collapsed relevant BRSA-A items into three domains as exposures of interest—suicidality, likelihood of accidental overdose and unsafe injecting practice. The suicidality domain consisted of suicide attempt history, suicidal ideation, carer concern and major mental illness items. The likelihood of accidental overdose domain consisted of reduced tolerance, recent abstinence, alcohol abuse and poly-substance use. The unsafe injecting domain included previous/current injecting, high risk injecting, and sharing of injecting equipment items. A score of 1 was assigned if any item within a given risk domain was scored as present; or 0 if all items within that risk domain were scored as absent—this increased power for all-cause and cause-specific overdose investigations. We chose to focus on these three domains as exposures because of their likely impact relationship on mortality in this patient group (World Health Organisation, 2013). Remaining BRSA-A items were included in analyses individually, as potential confounders.

### 2.6 Statistical analysis

Having checked proportional hazards assumptions, Cox regression (Cox, 1972) survival analyses were used to model the associations between the suicidality, accidental overdose, unsafe injecting domains (obtained from the first BRSA-A assessment in the observation period) and all-cause mortality. Competing risk regression was performed to model cause-specific overdose deaths for the same domains. Patients’ ‘at risk’ periods commenced from the date of their first BRSA-A assessment within the observation period (between 1 April, 2008 to 31 March, 2014) and ended on the day of their death or the end of observation period, whichever came first. We used likelihood ratio tests to examine potential interactions between risk domains and admissions to SLaM services in the two-month period after the assessment was conducted, and between risk domains and the OST prescriptions in the same observation period. Where a significant interaction was found we stratified the data accordingly and re-ran the Cox models with all-cause mortality as the outcome. Kaplan–Meier survival curves were used to visualize results for stratified analyses. All analyses were conducted using STATA 12, with significance levels at p < 0.05.

## 3 Results

The total number of patients with primary or secondary ICD10 F11 OUD diagnosis within the six-year period between 1st April, 2008 and 31st March, 2014 was 5335 and BRSA-A was completed for 84.1% (n = 4488) of those. There were no significant differences between age (calculated at midpoint observation period for this comparison), gender, ethnicity and mortality in people with and without completed BRSA-A assessments. There were no individual missing items within the group who had the BRSA-A completed. Therefore, the total number of individuals who met the inclusion criteria and whose data were extracted for analysis were 4488 (71.8% male; 66.9% “White British”), with 227 registered deaths (detailed in Table 1). Patients contributed a total of 17,804.59 at-risk person years. Age at risk assessment within our observation period ranged from 15 to 73 years with a mean age of 37.6 (SD = 9.07), and with mean age at death of 43.7 (SD = 9.15). More than a quarter (27.4%) of our OUD cohort were found to have a comorbid major mental illness. Majority of patients (64.2%) were admitted into SLaM services in the subsequent 2 months after their risk assessment was carried out.

Associations between suicidality, accidental overdose and unsafe injecting BRSA-A risk domains and all-cause mortality are represented in Table 2. In the fully adjusted models with all-cause mortality as an outcome, we found that BRSA-A assessed unsafe injecting and likelihood of accidental overdose was associated with increased risk of all-cause mortality (HR 1.53, 95% CI 1.10–2.11; HR 1.48, 95% CI 1.00–2.19 respectively).

We were able to obtain data on recorded underlying cause for 68.7% of deaths in our cohort (156/227), with overdose deaths (both accidental and intentional) being the largest group (n = 44). Other predominant causes of deaths within this cohort were deaths from hepatic causes (n = 39) and infectious diseases (n = 35) (data not shown in tables). In the fully adjusted competing risk regression models we found that BRSA-A assessed suicidality and unsafe injecting risks were independently and significantly associated with increased overdose mortality (sub-distribution hazard ratio [SHR] 2.88, 95% CI 1.38–6.03; SHR 2.52, 95% CI 1.11–5.67 respectively). Likelihood of accidental overdose was not associated with fatal overdose in these analyses.

In view of the significant findings above, we tested for the presence of interactions between admission in the 2-month period immediately after BRSA-A assessment and (1) suicidality, (2) accidental overdose and (3) unsafe injecting domains, in models where the outcome was all-cause mortality. An interaction between BRSA-A suicide risk and SLaM admission was found. Additionally, in all-cause mortality models, we tested for interactions between the types of opioid substitute treatment (i.e., buprenorphine, methadone, Suboxone [buprenorphine/naloxone]) and the three BRSA-A risk domains mentioned above but none were found (data not in tables)

**Fig. 1:**
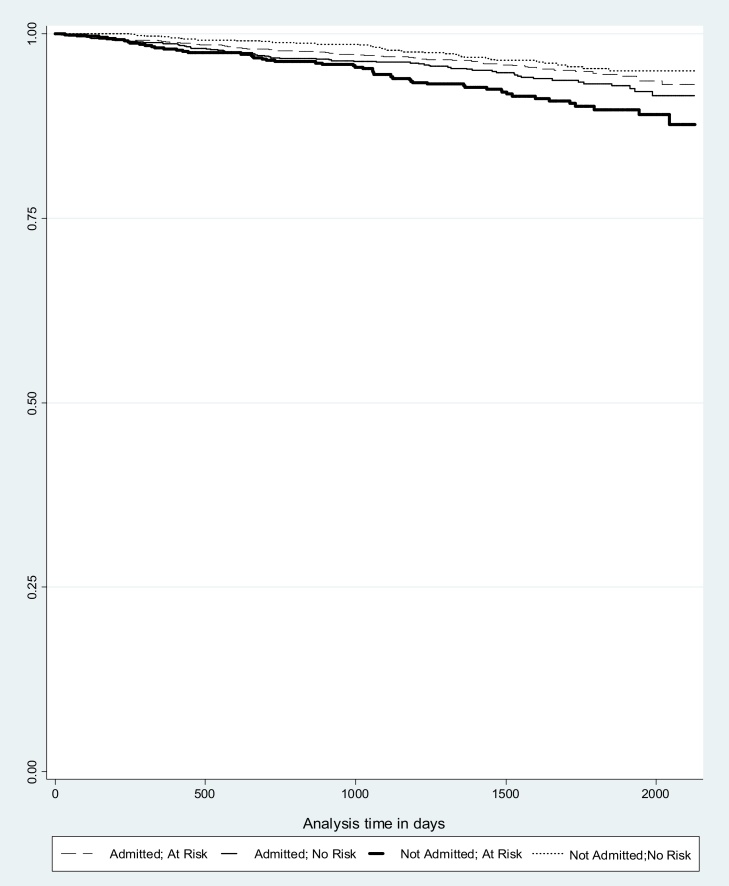
Kaplan–Meier survival curve for BRSA-A suicidality domain and admissions to SLaM services (in days).

After stratifying the analysis by admission to SLaM services (presented in Table 3) we found that an association between BRSA-A suicidality and all-cause mortality was present in the group who had not been admitted into SLaM services in the two months after their risk assessment (HR 2.03, 95% CI 1.67–3.24), but not for the admitted group. The Kaplan–Meier survival curve in Fig. 1 visualizes results for suicide risk domain stratified by admission to SLaM service showing the reduced survival in BRSA-A patients where suicidality was assessed as being present who were not admitted. Of all those admitted, 65.9% were admitted to addiction services, with other most common admissions being to psychological medicine and psychosis departments (data not shown in tables).

To establish the cause of non-admission, a manual search (where all free-text clinical notes and correspondence were reviewed) in the electronic patient records was conducted in a random sample of 200 patients who were not admitted to services in the 2-month period after their risk assessment (n = 100 where suicidality was assessed as being present in their BRSA-A; n = 100 where suicidality was not evident). Of those where suicidality was classified as being present, a manual electronic patient data search revealed that the leading causes for non-admission were loss of contact with the patient (51%) and transfer out of services (26%). Similarly, in the sample where suicidality was not evident, the leading cause for non-admissions were loss of contact with the patient (48%), transfer out of services (22%) and incarceration (11%). No interactions between BRSA-A risks of unsafe injecting and likelihood of accidental overdose and admission to services were found.

## 4 Discussion

Three important findings arising from this study ought to be noted. First, addiction-specific brief risk screen assessment may provide useful information to identify subgroups at elevated risk of mortality. Second, specific domains within the BRSA-A were particularly informative. Suicidality was found to be associated with increased risk of overdose mortality; unsafe injecting practices were associated with both all-cause and overdose mortality; and increased likelihood of accidental overdose was associated with all-cause mortality but not fatal overdoses. Finally, suicidality was associated with a twofold increased all-cause mortality risk among OUD patients who were not admitted to mental health services within 2 months of their risk assessment. However, we found no evidence that suicidality presented a similar risk in the subgroup who were admitted into mental health services during this time frame. These finding suggest that OUD patients with clinically evident suicidality who are not admitted to mental health services promptly may be particularly vulnerable

Whilst the relationship between drug injecting practices and increased all-cause and overdose mortality in OUD is consistent with current literature (Degenhardt et al., 2011; WHO, 2013), the relationship between overdose, suicide and intent is not as clear. Several studies have questioned to what extent heroin overdoses are de facto suicide attempts. An association between heroin overdose and suicide was noted, for example, in a study of 77 overdose survivors admitted to accident and emergency, with 49% reporting suicidal thoughts or feelings immediately prior to overdose (Neale, 2000). In another study among a London treatment sample, 50% of those with a history of overdose had two attempted suicides compared to 18% of those with no history of overdose (Vingoe et al., 2009). However, Darke and Ross, (2000) reported that while 40% of methadone maintenance participants had attempted suicide, only 10% had done so by means of a deliberate heroin overdose. Drug overdose was the most common method of attempted suicide, but by means of non-opioid pharmaceutical preparations. Conversely, heroin overdose among their participants overwhelmingly appeared to be accidental (92%).

Our data suggest that screening positively on at least one item within the suicidality domain, including suicide attempt and/or ideation, carer concern or major mental illness is, independently of accidental overdose risk factors, associated with an almost three-fold increase in fatal overdose. Although we do not know whether fatal overdoses in our cohort were indeed caused by heroin, other drugs, or a mixture of the two, it is noteworthy that in 2014 in England and Wales, more than a half of all deaths from drug poisoning involved an opiate drug (ONS, 2015). Second, because intent was unknown, we do not know which overdose deaths in our cohort were accidental and which were suicides. However, we did find an association between suicidality and overdose fatalities and did not find associations between increased likelihood of accidental overdose and overdose fatalities. This could be interpreted either that most overdose fatalities were deliberate (suicides), or that identification of patients as ‘likely to accidentally overdose’ resulted in higher visibility to services which then resulted in improved healthcare. Increased likelihood of accidental overdose may be addressed within addiction services, for example, by overdose training or supply of naloxone antidote. However, suicidality may be much more complex and problematic to address and with the need for dual-diagnostic/multidisciplinary care plan approaches addressing high levels of underlying depression and other psychiatric comorbidities (Bogdanowicz et al., 2015, Cantor et al., 2001, Darke et al., 2007).

The elevated mortality risk in patients where suicidality was evident and who were not admitted to mental health services in the subsequent two months, highlights the importance of admission, access to services and treatment provision. McCowan et al. (2009) describe history of admission as being a risk factor for mortality in this patient group. However, our study suggests that timing of admission itself is a protective factor for those at risk. Furthermore, non-admission into services was largely due to loss of contact and transfers out of service/catchment area. Drop-out from treatment (and relapse) and erratic engagement in services appears to be highly prominent in this patient group, and both are known to increase mortality considerably (Degenhardt et al., 2011, Zanis and Woody, 1998). Similarly, times of transition between services involved in the care of people with opioid dependency are particularly ‘risky’, for example after release from prison (Merrall et al., 2010). OUD patients who are assessed as being at risk of suicide and subsequently disengage with current services may require more determined strategies for patient follow-ups and service transition due to their high risk of mortality. Without better outreach for these poorly engaging groups, current policy will broaden inequalities for more vulnerable groups.

The results of this study need to be considered in light of certain limitations, alongside acknowledgement of strengths. SLaM is a large provider of secondary mental healthcare in Europe, with close to 100% monopoly provision to its geographic catchment. As a result, we were able to draw on electronic addictions service clinical records of almost five thousand OUD patients providing the statistical power to simultaneously control for a range of potential confounders. The inclusion criteria specified primary or secondary OUD diagnosis. Whilst the use of NLP applications allowed us to supplement the existing structured fields, it did not allow to us establish whether these diagnosis were primary or secondary and measure its impact on outcomes.

SLaM patient death-tracing is regularly updated and is based on death certificates issued across the UK for both active and non-active SLaM patients. This is not the case for underlying cause of death, which derives from additional static ONS linked data. Information on underlying cause of death was only present in 69% of cases. Additionally, as discussed, we could not differentiate between intentional (i.e., suicide-related) and non-intentional (i.e., accidental) overdose deaths. Similarly, toxicology reports were not available, and it was therefore unclear which drugs were involved in overdose deaths.

The clinical risk assessment information used for analysis was the first within the observation period but may not have been the first risk assessment conducted in an individual’s lifetime. Given the mean age of our cohort as 37, there will be individuals who have had previous treatment episodes, and subsequently previous risk assessment conducted, occurring prior to our observation period. Similarly, we do not know if any and which circumstantial/treatment changes occurred in the period beyond the subsequent two months after their risk screen and until their death/end of observation period, which might have influenced mortality risk in addition to clinically appraised suicide risk. However, given that a high proportion of people did not enter treatment due to loss of contact, it seems that the combination of suicidality and erratic engagement in services increases mortality in the longer term.

It is important to note that our analysis investigated admissions to mental health services across SLaM, and not addictions only. We chose to broaden our focus because suicide risk in OUD may not necessarily be attended to within the addiction setting in the first instance, especially in cases of psychiatric comorbidity. The identification of reasons for non-admission was extracted from a random sample and not the entire non-admitted sub-cohort. Although the administration of BRSA-A assessments is mandated in practice, only 84% of OUD patients had the BRSA-A scale completed. Finally, more consideration has to be given to the brief risk assessment screen as a measure of exposure status, which has advantages and disadvantages. The BRSA-A was not formally evaluated as a measurement in terms of constructs such as inter-rater or test-retest reliability, or its discriminant validity. However, this is a real-world measure, developed by clinicians and is actively used in daily practice, representing valuable and current real-life scenarios.

Prompt identification of those at risk is key. Our study provides evidence that addiction-specific risk assessment may be useful in predicting mortality in a timely manner. The study also points out associations between suicidality and overdose mortality in people with opioid dependency, and highlights the importance of admission to mental health services for those where suicidality is evident. Prompt identification and management of those at risk using brief risk assessment may be useful to save time, save costs and, most importantly, to save lives.

## Conflict of interest

RH and RS have received research funding from Roche, Pfizer, J&J and Lundbeck. JS has conducted a variety of research studies of addictive disorders and treatments and through the university, has provided consultancy to pharmaceutical companies about new treatments for opioid addiction, including Martindale, MundiPharma and Braeburn (none was the subject of study in this work). For further description see:

www.kcl.ac.uk/ioppn/depts/addictions/people/hod.aspx. Other authors have no conflict of interest.

## Contributions

All the authors listed contributed to the process of hypothesis generation, data collection, statistical analyses, or manuscript preparation, and fulfilled the criteria for authorship. Additionally, KMB wrote the manuscript and analyzed data. RDH and JS were primary supervisors. RS was responsible for securing funding for CRIS and constituent studies. MZ, JD, CKC (and all other authors) provided their suggestions during analysis and manuscript review process. HS is responsible for data extraction for analysis.

## Figures and Tables

**Table 1:** Cohort characteristics.

Variables	Number of individuals	Number of deaths (% per row)
** Total**	4488	227 (5.1)
**BRSA-A items and domains**		
**Suicide**		
Suicide attempt history	1279	91 (7.1)
Suicide ideations	306	13 (4.2)
Carer concern	205	17 (8.3)
Major mental illness	1225	75 (6.1)
**Accidental Overdose**		
Reduced tolerance	738	47 (6.4)
Recent abstinence	823	41 (5)
Alcohol abuse	1220	109 (8.9)
Poly-substance	2615	155 (5.9)
**Injecting**		
Previously injecting	1433	102 (7.1)
Currently injecting	1047	81 (7.7)
High risk injector	515	49 (9.5)
Share injecting equipment	367	32 (8.7)
**Violence**		
Violent past	1051	45 (4.3)
Violent thoughts	84	5 (6)
Violent Behaviour	119	8 (6.7)
Violence Concern	117	10 (8.6)
**Health BRSA Items**		
BBV Infections	900	92 (10.2)
Hist of s.rel.sezures	588	59 (10)
Unmet needs	717	92 (12.8)
Cognitive impairment	220	24 (10.9)
High risk sexual behaviour	258	14 (5.4)
**Social BRSA Items**		
Homeless/unstable housing	1341	76 (5.7)
Childcare/social service problems	392	17 (4.3)
social isolation	1246	88 (7.1)
self-neglect	816	74 (9.1)
criminal activity	1037	47 (4.5)
**Service Use Items**		
Erratic engagement	880	56 (6.4)

**Socio-demographic variables**		
**Age at assessment**		
15–24	358	9 (2.1)
25–29	614	13 (2.1)
30–34	833	36 (4.3)
35–39	888	47 (5.3)
40–44	869	45 (5.2)
45–49	536	33 (6.2)
50+	390	44 (11.3)
**Gender**		
Males	3224	166 (5.2)
Females	1264	61 (4.8)
**Ethnicity**		
White British	3002	170 (5.7)
Other White	622	32 (5.1)
Black	466	15 (3.2)
Mixed, unknown & other	398	10 (2.5)
**Level of deprivation (in tertiles)**		
Low (2.19–27.42)	1468	67 (4.6)
Moderate (27.43–37.0)	1470	77 (5.2)
High (37.1+)	1474	82 (5.6)

**Table 2:** Fully adjusted Cox and competing risk regression models examining associations between all-cause and cause-specific mortality and BRSA-A appraised suicidality, likelihood of accidental overdose and unsafe injecting in patients with opioid dependency.

Risk Cluster	Fully adj.^a^ all-cause HR (95% CI)	p value^a^	Fully adj.^a^ SHR for overdose^b^ deaths (95% CI)	p value^a^	Fully adj.^a^ SHR for deaths other than overdose (95% CI)	p value^a^
Suicidality						
None detected	Reference		Reference		Reference	
Detected (n = 1929, 120 deaths)	1.23 (0.92–1.64)	0.154	**2.89 (1.38–6.03)**	**0.005**	0.83 (0.55–1.26)	0.378
Likelihood of Accidental Overdose						
None detected	Reference		Reference		Reference	
Detected (n = 3416, 194 deaths)	**1.48 (1.00–2.19)**	**0.049**	2.82 (0.83–9.62)	0.097	1.23 (0.73–2.08)	0.43
Unsafe Injecting						
None detected	Reference		Reference		Reference	
Detected (n = 2249, 161 deaths)	**1.53 (1.10–2.11)**	**0.011**	**2.52 (1.11–5.70)**	**0.027**	1.37 (0.83–2.29)	0.221

Statistically significant (p < 0.05) hazard ratios are in bold

HR, hazard ratio; CI, confidence interval; SHR, sub-distribution hazard ratio.

a Adjusted for all variables listed in Table 1.

b Accidental and intentional overdoses.

**Table 3:** Cox regression analyses examining associations between suicide risk domain and all-cause mortality in individuals with opioid use disorder stratified by post BRSA-A admission to SLaM services.

	Hazard Ratio (95% CI), P value			
	Crude HR (95% CI)	p value	Fully adjusted^a^ HR (95% CI)	p value^a^
Not admitted (N = 1602, 90 Deaths)				
No suicidality detected	Reference		Reference	
Suicidality detected (n = 631)	**2.37 (1.56–3.62)**	**<0.001**	**2.03 (1.67–3.24)**	**0.003**
Admitted (N = 2881, 137 Deaths)				
No suicidality detected	Reference		Reference	
Suicide risk detected (n = 1294)	1.27 (0.91–1.78)	0.162	0.91 (0.63–1.32)	0.636

HR, hazard ratio; CI, confidence interval.

Statistically significant (p < 0.05) hazard ratios are in bold.

a Adjusted for all variables listed in Table 1.
